# Drought tolerance in soybean: genetics, metabolomics, remote sensing, and breeding for enhanced drought tolerance

**DOI:** 10.3389/fpls.2026.1733525

**Published:** 2026-03-13

**Authors:** Nathaniel Burner, Donna K. Harris, Clement Kwarteng Nyam, Ilyas Ahmad, Namhee Lee, Joon Hyuk Suh, Benjamin Fallen, Zenglu Li

**Affiliations:** 1 Department of Crop and Soil Sciences, and Institute of Plant Breeding, Genetics, and Genomics, University of Georgia, Athens, GA, United States; 2 Department of Plant Sciences, Sheridan Research & Extension Center, University of Wyoming, Sheridan, WY, United States; 3 Department of Food Science and Technology, University of Georgia, Athens, GA, United States; 4 Soybean & Nitrogen Fixation Research Unit, United States Department of Agriculture, Agricultural Research Service (USDA-ARS), Raleigh, NC, United States

**Keywords:** artificial intelligence, canopy wilting, drought tolerance, genomic prediction, high-throughput phenotyping, metabolomics, proteomics, QTL mapping

## Abstract

Drought is the most damaging abiotic stress affecting soybean production, with variable rainfall contributing significantly to year-to-year yield variability. Breeding efforts aim to develop cultivars with stable and competitive yields under both drought and non-drought stressed conditions. However, drought tolerance in soybean is a highly complex trait, influenced by diverse physiological, morphological, genetic factors and environments. Identifying genotypes with improved drought tolerance is challenging because traditional phenotyping methods for drought tolerance are subjective and time-consuming. Furthermore, quantitative trait loci (QTLs) associated with drought tolerance typically exhibit small effects and limited consistency across environments and populations. These challenges highlight the need for improved methodologies to identify and evaluate promising sources of genetic variation. This review summarizes the current state of drought tolerance breeding in soybean and discusses recent advances in remote sensing, transcriptomics, proteomics, and metabolomics aimed at enhancing drought tolerance research and cultivar development in soybean.

## 1 Introduction

Soybean [*Glycine max* (L.) Merr.] is the fourth most widely cultivated grain crop globally, following maize, wheat, and rice, with total production reaching approximately 378 million metric tons (Food and Agriculture Organization of the United Nations, 2024). However, soybean by far accounts for the largest share of global oilseed production, representing 59% of the total (American Soybean Association, 2024). In the 2023/2024 marketing year, the United States was the second-largest soybean producer after Brazil, contributing 31% of global production (113.3 million metric tons), with a market value of $60.7 billion (American Soybean Association, 2024).

Soybean is the leading agricultural export of the U.S. (USDA, 2022), with whole soybeans accounting for 75% of U.S. soybean exports, followed by soybean meal (20%) and oil (4%). Globally, soybeans contribute 69% of protein meal consumption (241 million metric tons). Soybean oil is the second most consumed vegetable oil behind palm oil (35%). Within the U.S., soybean is economically critical. It ranks second only to corn in production value. As expected, this economic impact is concentrated in the midwestern states that lead the United States in soybean production: Illinois, Iowa, Minnesota, Indiana, and Nebraska. These five states account for half of all soybean production in the United States (USDA, 2024).

Soybean is the subject of substantial private and public sector breeding efforts due to its importance. From 1990 to 2023, planted soybean hectarages in the United States have increased from 23.4 to 33.8 million hectares (American Soybean Association, 2024). During the same period, soybean yields increased from 2.22 to 3.46 metric tons per hectare, representing an average genetic gain of 14.4 kg per hectare annually (ASA, 2024). Soybean yield increases are attributable to the development of improved cultivars, improved management practices, and favorable interactions between these two factors (Rincker et al, 2014).

Yield evaluations of MG II-IV cultivars released between 1923 and 2008 indicated that soybean yields increased on average by 29 kg ha^-1^ yr^-1^ (Rincker et al., 2014). In this study, newer cultivars yielded more across both high- and low-yielding environments compared to older cultivars, although they exhibited less stability across environments. These results indicated that the yield increases of newer cultivars were partially attributable to being able to take advantage of higher yielding environments. A similar evaluation was conducted for cultivars in later MGs (V-VII) in the southern US released during a similar timeframe as the aforementioned study (Boehm et al., 2019). The estimated genetic gain was 13.7 kg ha^-1^ yr^-1^ after adjustment for maturity. The stability analysis in this study was consistent with that described by Rincker et al. (2014), in that newer cultivars exhibited greater responses in favorable production environments than older cultivars (Boehm et al., 2019).

Despite documented genetic gains, drought stress remains a major yield-limiting factor in soybean, and the inherent complexity of drought tolerance has continued to constrain breeding progress. While previous reviews have extensively examined soybean drought physiology or individual omics approaches in isolation (Arya et al., 2021; Chen et al., 2025; Hossain et al., 2013; Huang, 2025; Kumar et al., 2021; Purcell and Specht, 2004; Satpute et al., 2021; Tang et al., 2024), few have provided a comprehensive synthesis that integrates multi-omics approaches (QTL mapping, transcriptomics, proteomics, and metabolomics) with high-throughput phenotyping, remote sensing, and modern breeding strategies. As a result, a persistent gap remains between basic molecular discovery and its effective translation into drought-tolerant cultivars, particularly under variable field conditions. This review aims to address these gaps by exploring the links between genetics, physiological mechanisms, and emerging technologies. Specifically, it provides a unifying perspective on how integrating multi-omics, genomic prediction, artificial intelligence, and remote sensing can overcome traditional phenotyping bottlenecks and improve selection efficiency. By linking mechanistic understanding with applied breeding tools, this review offers a framework for accelerating the development and deployment of drought-tolerant soybean cultivars.

## 2 Impact of water stress on soybean growth and development

Drought is widespread, affecting approximately 55% of the contiguous United States’ land area in early 2021 (National Drought Mitigation Center (NDMC), 2021). It contributed to an estimated 36% reduction in yield (Specht et al., 1999). Soybean plants experience drought stress when water availability is insufficient to meet their transpiration and evapotranspiration demands (Rauf et al., 2016). Physiologically, this stress typically manifests when leaf osmotic potential drops below a critical threshold of approximately -1.1 to -1.2 MPa, leading to a rapid decline in leaf enlargement and net photosynthesis. In field and greenhouse conditions, these potentials are often reached when soil moisture availability falls below 50–60% of field capacity or total available water (Nagasuga, 2019; Nielsen, 1990). This can occur due to prolonged periods without rainfall or irrigation, typically ranging from a few days to several weeks depending on the growth stage. For instance, during the critical reproductive stages (R3 to R6), even four days of visible moisture stress can lead to significant yield reductions of 39–45%. These periods are often characterized by high temperatures exceeding 35 °C (95°F) and a high vapor pressure deficit (VPD > 2.0 kPa), which accelerates soil moisture depletion and increases the plant’s transpiration demand (Fletcher et al., 2007). Crop losses due to drought resulted in billions of dollars in insurance indemnities—more than any other abiotic or biotic stress (Boyer, 1982; Khedun and Sohoulande, 2025; Wilhite et al., 2007). From 1980 to 2021, drought accounted for 9% of weather-related events, causing at least $1 billion in damages (NOAA, 2022). In the United States, only 16% of harvested soybean acreage is irrigated (USDA, 2024), leaving most production vulnerable to drought.

Water stress alters soybean phenology and reduces overall plant performance (Desclaux and Roumet, 1996). It also reduces the number and rate of appearance of new mainstem nodes and hastens the phases of reproductive organ development on structures produced during and after stress. Determinate and indeterminate soybeans differ in the prioritization of reproductive structures in response to stress, with the former tending to preserve those on the branches and the latter preserving those on main stem nodes (Desclaux and Roumet, 1996). Among the reproductive structures found on either branches or main stems, soybean plants tend to prioritize older reproductive structures in response to water stress. However, drought during pod formation and seed filling increases seed abortion (Desclaux and Roumet, 1996). As a result, water stress that occurs in these later stages has the most detrimental effects on seed yield (Sionit and Kramer, 1977).

Plant cells must maintain sufficient turgor pressure to perform essential physiological functions (Boyer, 1970). Turgor pressure occurs when the aqueous system within a plant’s cells exerts a positive pressure on the cell walls, resulting in an upright and erect plant. Sufficient upward water movement from the soil to the foliar tissue is required due to the differences in total water potential throughout the plant to maintain a positive turgor pressure (Blum, 2017). In plants, total water potential is the difference in potential energy between two aqueous systems. Water moves from areas of higher to lower potential; thus, turgor is maintained when external water potential exceeds that within cells (Cortes and Sinclair, 1986). Wilting occurs when leaf cells lose water because the leaf water potential exceeds that outside the cells.

The rates of leaf enlargement and net photosynthesis in whole soybean plants (cv. Haro soy) were shown to rapidly decline when leaf water potential fell below a minimum threshold in response to soil desiccation (Boyer, 1970). Specifically, leaf enlargement was inhibited once leaf water potentials dropped to approximately −4 bars (−0.4 MPa), while net photosynthesis remained unaffected until potentials reached roughly −11 bars (−1.1 MPa). At leaf water potentials between −14 and −15 bars, the photosynthetic rate was reduced by 50%. This demonstrates that the rapid decline in leaf enlargement occurs at a significantly higher leaf water potential than for photosynthesis, indicating that vegetative growth is more sensitive to water deficit than carbon assimilation (Boyer, 1970). The author also proposed that the loss of leaf water potential promoted a plant to translocate water away from sites of growth because the reduction of leaf enlargement did not appear to be due to a lack of photosynthates.

## 3 Advancement in drought tolerance research

### 3.1 Physiological mechanisms underlying canopy wilting

The most visible symptom of drought stress in soybean is the loss of leaf turgor, which leads to drooping and the upward flipping of the abaxial surface of leaves, a phenomenon referred to as canopy wilting (CW) (King et al., 2009; Ries et al., 2012; Sloane et al., 1990). This flipping exposes the lighter underside of the leaves, increasing sunlight reflectance and reducing canopy temperature (Casteel, 2012). Drought-stressed soybean plants also clamp their leaves inward to reduce the amount of leaf surface area exposed to sunlight, thereby reducing photosynthetic activity (Edwards, 2021). The onset and severity of CW vary by genotype, and in general, genotypes that exhibit delayed CW tend to suffer less yield loss under drought-stressed conditions compared to those that exhibit fast wilting (Sloane et al., 1990; Ye et al., 2020).

However, the slow CW phenotype is not soybean’s primary defense mechanism against drought; instead, it is the culmination of a number of other beneficial defense mechanisms such as increased water-use efficiency, greater access to soil moisture, and reduced transpiration rates (Ludlow and Muchow, 1990; Purcell and Specht, 2004). A plant’s yield under drought stress conditions is a function of three mostly independent components: plant transpiration, water-use efficiency, and harvest index (Passioura, 1977). Several traits and physiological mechanisms in soybean have been observed or theorized to have substantial influence over these components (Ludlow and Muchow, 1990; Purcell and Specht, 2004). Accordingly, no single trait accounts for the delayed CW phenotype.

The ability of a soybean genotype to conserve soil water content has been identified as strongly correlated with its CW phenotype during drought-stressed conditions (King et al., 2009; Ries et al., 2012). Ries et al. (2012) demonstrated that contrasting phenotypic profiles in soybeans can lead to differential soil water conservation, which in turn delays the onset of CW. The authors initially hypothesized that slow CW genotypes would exhibit a combination of low radiation use efficiency (RUE) and high water-use efficiency (WUE), thereby reducing transpiration rates and conserving water. RUE refers to the ratio of biomass gain to accumulated photosynthetically active radiation intercepted by a plant, while WUE is the ratio of increased biomass to transpired water during a given period of time (Ries et al., 2012). However, high or low values of either metric were not found to be exclusive to a specific wilting class. The authors concluded that genotypes exhibiting both high WUE and RUE could have higher rates of photosynthesis due to low internal levels of CO2 (Ries et al., 2012). In contrast, genotypes exhibiting both low WUE and RUE achieved higher soil water conservation through lower stomatal conductance but suffered from a low yield potential (Ries et al., 2012).

The transition from healthy soybean growth to canopy wilting is defined by a cascade of physiological and biochemical shifts that occur as soil moisture depletes. Research by Chowdhury et al. (2016) and Fatema et al. (2023) provides a quantitative framework for these responses, showing that soybean begins to exhibit significant stress as leaf water potential drops toward a critical threshold of approximately −1.1 to −1.2 MPa, at which point net photosynthesis (Pan) typically ceases. This decline is preceded by a rapid reduction in stomatal conductance (gas), which often falls from a well-watered baseline of 0.30 to as low as 0.03 mol m^−2^ s^−1^, leading to a roughly 40% reduction in photosynthetic rate. Concurrently, the maximum photochemical quantum yield (Fl/Fm) decreases from 0.80 to below 0.70, signaling early damage to photosystem II. To maintain cellular integrity during these shifts, soybean employs osmotic and antioxidant defenses. Tolerant genotypes typically exhibit a significant increase in proline (often exceeding a five-fold increase) to facilitate osmotic adjustment, while sensitive lines show sharp spikes in malondialdehyde (MDA), a marker of lipid peroxidation, rising from ~7 nmol/g to over 70 nmol/g. To mitigate this oxidative damage, the plant upregulates antioxidant enzymes such as superoxide dismutase (SOD) and catalase (CAT), which generally show an activity increase of 15% to 30% during moderate stress before potentially declining as drought severity becomes terminal.

### 3.2 QTL mapping and gene discovery for improved drought tolerance

The slow CW trait in soybean is highly polygenic, reflecting the complex interplay of physiological mechanisms that may contribute to it (Charlson et al., 2009; Menke et al., 2024; Burner et al., 2025). Due to the quantitative nature of the trait, the underlying genetic mechanisms controlling it remained elusive until the availability of dense genetic marker maps and the advent of more efficient genotyping platforms (Valliyodan et al., 2017). Charlson et al. (2009) identified four QTLs on chromosomes (Chrs) 8, 13, 14, and 17 from an F_5_–derived RIL population derived from a cross of the drought-sensitive ‘KS4895’ (MG IV) × drought-tolerant ‘Jackson’ (MG VII). Only the QTL on Chr 13 was identified across two environments; however, a QTL on Chr 17 explained the highest proportion of phenotypic variation across environments (15%) (Charlson et al., 2009). Interestingly, only one of the four slow-wilting QTL alleles was derived from ‘Jackson’, likely due to the parental lines being selected for contrasting N_2_ fixation under drought, rather than CW (Charlson et al., 2009). Most of the QTLs were associated with QTLs for other traits, including seed protein and oil content, corn earworm and Javanese root-knot nematode resistance, and aquaporin production (Charlson et al., 2009).

Eight QTLs were identified from a ‘Kefeng1’ × ‘Nannong1138-2’ RIL population, of which two were identified under both field and greenhouse conditions (Du et al., 2009). A QTL identified on Chr 8 explained the highest proportion of phenotypic variance (24%) (Du et al., 2009). Abdel-Haleem et al. (2012) detected seven CW QTLs across five site-years from an F_5_–derived ‘Benning’ × PI 416937 population (Boerma et al., 1997). These QTLs were located on Chrs 2, 4, 5, 12, 14, 17, and 19, and collectively explained 75% of the phenotypic variance for CW, with the QTL on Chr 12 accounting for the highest proportion of phenotypic variation (27%) (Abdel-Haleem et al., 2012). The QTLs on Chr 14 were identified in both Abdel-Haleem et al. (2012) and Charlson et al. (2009) studies, with the favorable CW alleles derived from ‘Benning’ and ‘Jackson’, respectively. ‘Jackson’ was the more drought-tolerant parent in the Charlson et al. (2009) study and is in the pedigree of ‘Benning’ (Abdel-Haleem et al., 2012). Only the Chr 12 QTL was detected in all site-years and may be co-localized with QTLs for silver nitrate response, yield under drought stress, oil content, iron efficiency, seed weight, and seed protein (Abdel-Haleem et al., 2012). In a previous study, five QTLs for fibrous roots were mapped in the same Benning × PI 416937 RIL population described by Abdel-Haleem et al. (2012). These fibrous root QTLs were located on Chrs 1, 3, 4, 8, and 20 and accounted for 7.3–13.5% of phenotypic variation. Of these, only the Chr 4 fibrous root QTL co-localized with the Chr 4 CW QTL identified by Abdel-Haleem et al. (2012).

Using introgressed lines that possessed four QTL for slow CW and two QTL associated with fibrous root QTLs (Abdel-Haleem et al., 2011, 2012), Burner et al. (2025) attempted to validate the effects of these QTLs on CW. However, the lines did not exhibit significantly reduced CW compared to the recurrent parents. Although not significant, a line possessing a Chr 12 introgression exhibited a 36–41% reduction in CW score under drought conditions. Hwang et al. (2015) evaluated five drought-sensitive × drought-tolerant RIL populations, including the two used by Charlson et al. (2009) and Abdel-Haleem et al. (2012). Eight QTL clusters were identified on Chrs 2 (3 QTLs), 5, 11, 17 (2 QTLs), and 19 (Hwang et al., 2015). Among these, only the Chr 17 QTL from Charlson et al. (2009) and the Chrs 2 and 5 QTLs from Abdel-Haleem et al. (2012) were consistently detected. However, many of the other QTLs appeared in separate clusters in single populations. The QTL on the Chr 14 cluster identified in three of the populations were flagged as potential false positives (Hwang et al., 2015). In a follow-up study, a meta-QTL analysis of the eight QTL clusters identified by Hwang et al. (2015) concluded that six meta-QTLs on Chrs 2, 5, and 17 would be useful for marker-assisted selection (MAS) due to their stability across years and populations (Hwang et al., 2016). The Chr 11 and 19 meta-QTLs were identified as major QTLs but were not consistently identified across populations and years (Hwang et al., 2016). Menke et al. (2024) mapped the CW trait in a Benning × PI 471938 RIL population evaluated across seven site-years. Three QTLs on Chrs 2, 8, and 9 were identified in the combined site-year analysis, explaining 10 - 14% of the phenotypic variation. All three QTLs were found in similar regions as CW QTLs identified by Hwang et al. (2016) and Abdel-Haleem et al. (2012) (Chr 2), Kaler et al. (2017) (Chr 8 and 9), and Steketee et al. (2020) (Chr 9). Six QTL identified in the study were significant in single-environment analyses but not in the combined analysis, further highlighting the instability of QTLs associated with CW.

PI 567690 and PI 567731 are unique among known slow CW accessions due to their early maturity group (MG III) (Pathan et al., 2014). This trait makes them potentially valuable for breeding drought-tolerant cultivars suited for early maturity groups. Two F_7_–derived RIL populations derived from ‘Pana’ × PI 567690 and ‘Magellan’ × PI 567731 led to the identification of eight and two QTLs for slow CW, respectively. The slow CW parent was the donor line for the favorable alleles for nine of the 10 QTLs (Ye et al., 2020). All eight QTLs from the ‘Pana’ × PI 567690 population had previously been reported. In contrast, neither of the QTLs detected on Chrs 6 and 10 from the ‘Magellan’ × PI 567731 had been previously reported. The QTLs on Chrs 5 and 6 explained the highest proportion of phenotypic variation at 10.4 and 29.6%, respectively. Recently, Burner et al. (2025) evaluated a Benning × PI 603535 RIL population under extremely arid conditions for three years and identified seven CW QTL on Chrs 2 (2 QTLs), 3, 7, 12, 13, and 19. Of these, the Chr 12 QTL explained the highest phenotypic variation (10.3%), which was located near a significant locus identified by Kaler et al. (2017).

Genome-wide association studies (GWAS) have also been utilized to further elucidate the complex genetic mechanisms underlying the slow CW trait, facilitated by recent advancements in genotyping technologies. Kaler et al. (2017) evaluated 373 MG IV accessions from diverse geographic origins and identified 185 genotypes with CW scores lower than PI 416937, detecting 51 loci associated with the CW trait. The minor allele of a SNP detected on Chr 18 was found to have the largest reduction in CW and associated with a gene encoding a protein involved in water transportation. Additionally, a SNP detected on Chr 6 was associated with a gene that encodes a protein in response to abscisic acid stimulus. Abscisic acid is a plant hormone involved in the signaling pathway in response to several stresses (Swamy and Smith, 1999). SNPs on Chrs 2 and 11 were associated with genes involved in root hair elongation and jasmonic acid biosynthesis. Overall, six loci with favorable minor alleles were located within the QTL clusters previously described by Hwang et al. (2016). Subsequently, using a panel of 200 accessions including 100 accessions from Kaler et al. (2017); Chamarthi et al. (2021) identified 188 significant SNPs associated with CW. Of those, 87 SNPs representing 68 loci, overlapped with those reported previously by Kaler et al. (2017).

A similar GWAS study of 162 MG VI–VIII accessions identified 78 genotypes with wilting scores lower than PI 416937 and 44 SNPs associated with CW (Steketee et al., 2020). Nine SNPs were in genomic regions overlapping with QTLs reported (Charlson et al., 2009; Abdel-Haleem et al., 2012; Hwang et al., 2015; 2016) and 12 significant loci were also found in the Kaler et al. (2017) study. A Chr 2 QTL contributed the greatest reduction in CW and was associated with an aldehyde dehydrogenase gene, whose expression is highly induced in soybean under drought conditions (Wang et al., 2017). Candidate gene models associated with two other significant loci were reported to be involved in drought stress responses.

While these biparental QTL mapping and GWAS have focused on adult plant drought tolerance primarily using the CW trait, several recent studies have shifted attention to drought tolerance at the seedling stages or seed germination date under controlled environments (Aleem et al., 2024; Kong et al., 2025; Zhang et al., 2022). Using a population of 234 RILs and a panel of 259 accessions phenotyped for the seedling survival ability at the V2 seedling stage, Zhang et al. (2022) identified nine and 53 QTLs across 19 chromosomes, respectively. A combination analysis of the two populations revealed two common QTLs. Similarly, Aleem et al. (2024) evaluate a panel of 240 accessions from 20 countries for germination rate, whole seedling length and root length under drought stress in control conditions, identifying 27 genomic regions associated with the three traits. Of those, a QTL on Chr 8 was found to be associated with both germination rate and root length. To investigate the genetic controls of seed germination in a diverse panel of 207 soybean accessions, GWAS detected 58 QTLs, 10 of which explained a relatively larger portion of phenotype variation (Kong et al., 2025). However, to date, no studies have been reported on the links of the drought tolerance between the seeding stage and adult plant stage. A table summarizing the QTLs identified is presented in **Supplementary Table 1**.

### 3.3 Transcriptomic analysis for drought tolerance

Drought tolerance in plants is mediated by complex physiological mechanisms stemming from cellular responses such as stomatal closure, accumulation of antioxidants and osmoprotectants, scavenging of reactive oxygen species, and changes in membrane stability (Liu et al., 2014; Dinneny, 2019; Hipsch et al., 2021). Transcriptomic analysis, aided by RNA sequencing (RNA-Seq), an approach that relies on next-generation sequencing, has enabled the high-throughput investigation of the transcriptome in plants. This technology has provided insights into the molecular pathways and genes that regulate abiotic stress response, leading to the discovery of candidate genes underlining drought tolerance in soybeans (Wang et al., 2009; Behjati and Tarpey, 2013; Cao et al., 2022).

Molinari et al. (2023) identified abscisic acid (ABA)-responsive, differentially expressed genes (DEGs) in a greenhouse hydroponic system using drought-tolerant (Embrapa 48) and drought-sensitive (BR16) soybean cultivars, which were grown under mild, moderate, and severe water deficit conditions. Water deficit conditions were achieved by moving plants from hydroponics into empty boxes. The duration after treatment, in minutes, represented the different treatments: 0 min for control, 25–50 min for mild, 75–100 min for moderate, and 125–150 min for severe water deficit. Of the identified genes, 134 were found in both soybean cultivars (drought-tolerant and -sensitive) and in both leaf and root tissues across the four different treatments. Of these genes, 125 were upregulated and nine were downregulated. These genes showed no variation in expression profiles across water deficit treatments. Root tissue showed more ABA-responsive genes and pathways triggered in response to water deficit conditions in both the drought- tolerant and -sensitive cultivars. When alternative splicing events from whole transcriptome RNA-seq data were analyzed by Song et al. (2020) in the William 82’s roots under different drought conditions (5 d withholding water for very mild stress, 12 d for mild stress, 19 d for severe stress, and water recovery where plants were rewatered for 2 d after severe water stress), 2120 genes were identified that experienced significant alternative splicing regulation. Among the alternative splicing events identified, the major types were alternative 3´ splice sites and skipped exons. In a seedling assay using two drought-tolerant (PI 342618B and A214) and two drought-sensitive (NN86–4 and A195) genotypes under controlled and drought stress conditions using polyethylene glycol (PEG), Aleem et al. (2021) identified 10 genes as possible candidate genes in an RNA-seq analysis. Eight of these identified genes were located on Chr 8 in the regions previously identified by Manavalan et al. (2015) and Valliyodan et al. (2017) to be hotspots associated with drought tolerance in soybean. Another soybean seedling assay focused on the soybean *bZIP* transcription factor, which has been reported to be crucial to abiotic stress response in plants. In this study, both salt and drought stresses were imposed on soybean C03–3 wild-type and transgenic lines. The transgenic soybean plants, expressing a functional repressor form of *GmbZIP15*, exhibited drought stress resistance similar to that of the wild-type C03–3 soybean, while overexpression of *GmbZIP15* resulted in hypersensitivity to the stress conditions compared to the C03–3 wild-type. Through RNA-seq analysis and qRT-PCR, it was shown that *GmbZIP15* positively regulates *GmSAHH1* expression and negatively regulates *GmWRKY12* and *GmABF1* expression in response to both salt and drought stress conditions (Zhang et al., 2020). In an RNA-seq profiling of leaf tissue from two soybean cultivars, SS2-2, a drought-tolerant cultivar, and Taekwang, a drought-sensitive cultivar, water was withheld at the V3 (BBCH: 12) stage to impose drought stress (Fehr et al., 1971; Earth Observation Research Branch Team, 2011). In this greenhouse study, the two cultivars assayed at the V4 (BBCH: 14) stage of development exhibited differentially expressed genes involved in lipid metabolism pathways in the drought-tolerant cultivar; however, in the drought-sensitive cultivar, the expression of these same genes remained unchanged (Yang et al., 2023).

Using RNA-seq, Bao et al. (2024) identified a salt stress-inducible gene, *GmRVE8a* from soybean. When it was transferred to Arabidopsis, three-week old transgenic plants grew significantly faster than the wild-type plant under both salt and drought stress conditions. Additionally, the malondialdehyde (MDA) content in the transgenic lines was significantly lower than that of the wild type. These results suggest that this gene may act as a positive regulator in responses to both salt and drought stress environments. Zhao et al. (2024) characterized the ankyrin repeat-TM subfamily (*ANK-TM*) protein and demonstrated that the overexpression of *GmANKTM21* in transgenic *35S:GmANKTM21* Dongnong50 soybean plants under drought stressed conditions in a controlled environment seedling assay exhibited lower MDA content, lower water loss, less reactive oxygen species production, and higher stomatal closure in soybean when compared to the wildtype. RNA-seq analysis of differentially expressed genes in the transgenic lines identified three genes (*GmSPK2*, *SmSPK4*, and *GmANKTM21*) that showed higher expression in the transgenic soybean than in the wild-type.

In a PEG-simulated drought stress experiment conducted by Wang et al. (2022c), the transcriptomic and metabolomic response of the drought-tolerant soybean cultivar ‘Heinong 65’ was compared to ‘Heinong 44’ a cultivar sensitive to drought. The transcriptomic and metabolomic analyses of these soybean seedlings revealed key candidate genes and metabolites that may be crucial to drought response, involved in the tricarboxylic acid cycle (TCA) cycle and isoflavone biosynthesis pathway. While most studies focusing on RNA-seq analysis of drought tolerance have been conducted during the vegetative stage of development, a study by Correa Molinari et al. (2021) examined sensitivity to drought in both flowering (R2, BBCH: 65) and pod-filling (R4, BBCH: 73) stages of development. The drought-sensitive cultivar ‘BR16’ was subjected to water deficit conditions at the V7 stage. Flowers and pods were collected at the R2 and R4 stage, respectively, for RNA extraction and analysis. RNA-seq analysis revealed that while most differentially expressed genes in flowering were downregulated, during pod fill the majority of differentially expressed genes were upregulated. The authors suggested that this difference in expression profile is likely due to drought conditions inhibiting gene expression during flowering, as soybeans are more sensitive to water stress. On the other hand, in the pod filling stage, there was upregulation of genes and pathways which could indicate the strategy of the plant to make seed, even in drought conditions, to preserve and transmit genetic material to the offspring, even if this results in loss of yield (Correa Molinari et al., 2021).

In a study by Wang et al. (2024a), the regulatory responses of ‘Liaodou 15’ to drought stress were analyzed. Liaodou 15 is a drought-sensitive soybean cultivar, and tissue for the analysis was obtained at the flowering stage under three different levels of drought (withholding irrigation for 7 d for mild stress, 17 d for moderate stress, and 27 d for severe stress) and a control in field conditions. Results indicated that drought stress induced the expression of *P5CS* and *PAO* genes (*PAO1*, *PAO4*, and *PAO5*) that promote the accumulation of spermidine and proline, compared to the response in the control treatment. Another transcriptome analysis study conducted at flowering using this drought-sensitive cultivar ‘Liaodou 15’ revealed differentially expressed genes involved in ABA and flavonoid biosynthesis as well as ascorbic acid and glutathione metabolism. Transcription factors such as *WRKY*, *MYB*, and *bZIP* were also identified, which may play a role in regulating the response to drought conditions (Li et al., 2022a). A mild water deficit greenhouse experiment was conducted at the seed filling stage (R5, BBCH: 75) using a drought-tolerant variety, Heinong 44’, and a drought-sensitive variety, ‘Suinong 14’, with results showing that the majority of the differentially expressed genes were upregulated and enriched in the oxidative stress response pathway in both varieties. However, the differentially accumulated metabolites were mostly upregulated in the α-linolenic acid metabolism and amino acid and nucleotide sugar metabolism pathways and downregulated in the histidine metabolism pathway in the drought-tolerant variety. Whereas, in the drought-sensitive variety, the α-linolenic acid metabolism and the lutein synthesis pathways were downregulated (Xu et al., 2024).

Transcriptomic studies have been employed to investigate the molecular mechanisms underlying slow canopy wilting in soybeans, particularly for PI 416937 (Shin et al., 2015) and PI 567690 (Prince et al., 2015). Slow wilting in soybean has been shown to be an important physiological mechanism in drought conditions and is characterized by a slow wilting of the canopy, resulting from a cellular process that enables reduced transpiration rates and plant water conservation during drought stress (Ye et al., 2020). In their comparative transcriptomic study, Shin et al. (2015) subjected Benning, a drought-sensitive cultivar, and PI 416937, a drought-tolerant landrace, to drought stress at the R2 stage. The authors reported changes in the transcriptomic response to drought in PI 416937 and Benning, including the downregulation of photosynthesis and the upregulation of protein transport and chromatin remodeling. They hypothesized that the slow-wilting trait of PI 416937 may be due to a decrease in the expression of genes associated with water transport in response to drought stress, thereby ensuring water retention through reduced transpiration. Li et al. (2024) also performed a comparative transcriptomic analysis using Liaodou 14, a drought-tolerant soybean cultivar with a slow-wilting phenotype, and Liaodou 21, a drought-sensitive cultivar, under PEG-induced drought stress. In this greenhouse study, soybean plants were grown in half-strength Hoagland solution, and drought stress was imposed at the V2-V3 stage using 20% PEG. Their results revealed six leucine-rich repeat receptor-like kinases (LRR-RLKs) as potential candidate genes for drought avoidance. Xu et al. (2023b) also reported in their comparative transcriptomic study of two contrasting genotypes that HN44 showed better drought-tolerance than SN14 through key DEGs such as *GmbZIP4*, *LOC100810474*, and *LOC100819313* in the major pathways. A summary of transcriptomic studies on soybean responses to drought stress conditions can be found in **Supplementary Table 2**.

Although several studies have been conducted in recent years to identify genes that are up- or down-regulated in response to drought stress, a paper by Sinha et al. (2023) highlights the complexity of utilizing these findings to breed soybean varieties with drought stress tolerance. In their study, transcriptomic data were analyzed from soybean leaf, pod, sepal, stigma, and overall tissue from three different treatments (water deficit, heat stress, and a combination of both), showing a differential expression of transcripts from each tissue within each of the different stress conditions, including the combination of the water deficit and heat stress treatment. These findings suggest that a more complex and coordinated approach to breeding for tolerance to different abiotic stress conditions may be necessary, where the expression of different groups of transcripts is altered simultaneously in various plant tissues through a stress-specific approach (Sinha et al., 2023). Shafiq et al. (2025) analyzed RNA-seq metadata using publicly available data that fell into three categories: drought, heat, and water stress. Results identified 398, 332, and 322 upregulated genes and 453, 301, and 455 down-regulated genes in response to drought, heat, and water stress, respectively. However, only four down-regulated genes were common between drought and water stress, and 14 were upregulated between these two categories, pointing again to the complexity involved in breeding for stress tolerance.

### 3.4 Proteomics in soybean drought tolerance

Drought stress has a strong impact on plant growth, physiology, and development, forcing plants to adjust their biochemical and molecular processes to survive under limited water availability. One of the most effective ways plants cope with drought is by changing the levels and activities of proteins and metabolites, which are often regulated by changes in gene expression. Although mRNA expression can provide useful information, it does not always accurately reflect protein abundance because many regulatory processes occur after translation, such as phosphorylation, ubiquitination, and protein degradation (Greenbaum et al., 2003; Hossain et al., 2013). As a result, direct analysis of proteins is essential to gain a true understanding of plant responses to drought stress (Mahmood et al., 2019; Singh et al., 2022).

Proteomics provides a powerful approach to study these protein-level changes and offers a comprehensive view of cellular responses under drought conditions. Several advanced proteomic techniques have been developed for this purpose, including two-dimensional difference gel electrophoresis (2D-DIGE), label-free quantification, and labeling methods such as iTRAQ and tandem mass tag (TMT) analysis (Cox et al., 2014; Wiese et al., 2007; Zecha et al., 2019). Among these, label-free quantification is widely used because of its efficiency and suitability for large-scale protein analysis (Clough et al., 2009; Lee et al., 2010). These techniques allow the identification of proteins that are induced or suppressed by drought, as well as proteins that undergo post-translational modifications, helping to uncover key pathways involved in stress tolerance. A large number of proteomic studies have examined soybean responses to various abiotic stresses, including drought (Alam et al., 2010; Toorchi et al., 2009), heat (Ahsan et al., 2010; Katam et al., 2020), salinity (Aghaei et al., 2009; Ji et al., 2016; Ma et al., 2012; Onishi et al., 2006), and cold stress (Cheng et al., 2010; Tian et al., 2015). In this review, the focus is specifically on proteomic responses of soybean to drought stress.

Drought stress strongly affects photosynthesis, one of the most sensitive processes in plants. Proteomic analyses have shown that drought alters the abundance of several photosynthesis-related proteins in soybean, leading to disturbances in RuBisCO activity, electron transport, and the Calvin cycle (Das et al., 2016). These disruptions limit carbon fixation and reduce plant productivity. However, drought-tolerant soybean genotypes are often able to maintain higher levels of photosynthetic proteins, allowing them to sustain photosynthesis under stress. S-adenosylmethionine is closely linked to photosynthetic performance due to its essential role in chlorophyll biosynthesis. Toorchi et al. (2009) analyzed the proteomic response of the soybean variety Enrei under PEG-induced osmotic stress and reported a reduction in S-adenosylmethionine synthetase abundance during drought conditions. In addition, they also observed caffeoyl-CoA O-methyltransferase and the 20S proteasome alpha subunit A showed contrasting expression patterns under osmotic stress, indicating their important involvement in the early root response to short-term drought stress.

Elongation factor Tu (EF-Tu) is one such protein that plays an important role in stress tolerance. In soybean cultivars ‘Surge’ and ‘Davison’, combined drought and heat stress resulted in higher EF-Tu abundance in the more tolerant cultivar Surge (Das et al., 2016). EF-Tu functions not only in protein synthesis but also as a molecular chaperone, supporting plastid translation, protein folding, and signaling pathways involved in heat stress responses (Bhadula et al., 2001; Fu and Ristic, 2010; Ristic et al., 2004). Overexpression of EF-Tu has been shown to improve heat tolerance in several crop species (Ahsan et al., 2010; Fu et al., 2012). However, under severe heat stress conditions, EF-Tu levels may decrease, as observed in soybean cv. Enrei at 40 ± 2 °C, indicating that extreme stress can impair protein synthesis mechanisms (Ahsan et al., 2010). Differences in drought tolerance among soybean genotypes are also linked to photosynthetic efficiency. A comparative proteomic study showed that the drought-tolerant genotype GN-3074 had higher levels of photosynthetic and oxidative stress defense proteins than the sensitive genotype GN-2032 (Yahoueian et al., 2021). Proteomic studies have also identified changes in enzymes related to redox regulation and antioxidant defense. Reduced expression of methionine synthase in soybean seedlings (cv. Enrei) was associated with improved drought tolerance through increased activity of antioxidant enzymes such as catalase (CAT), peroxidase (POX), and superoxide dismutase (SOD), along with higher proline and chlorophyll levels (Maqsood et al., 2022; Mohammadi et al., 2012). These responses help limit oxidative damage and support plant survival under drought conditions.

Enzymes involved in stress recovery also show dynamic changes. In soybean cv. Enrei, peroxidase activity decreased during drought but increased during recovery, whereas aldehyde dehydrogenase (ALDH) activity showed the opposite pattern, increasing during stress and declining after rewatering (Khan and Komatsu, 2016). Soybean contains a large ALDH gene family, with 53 GmALDHs genes identified and grouped into 10 families (Wang et al., 2017). Changes in protein abundance are particularly sensitive indicators of drought stress in soybean roots (Wang and Komatsu, 2017). In a proteomic study of the drought-tolerant soybean ‘Jiyu47’, TMT-based analysis revealed significant changes in proteins related to carbohydrate metabolism, osmotic regulation, and antioxidant defense under osmotic stress (Zhou et al., 2022). Among these, proteins involved in glutathione metabolism were strongly affected. Glutathione is a key metabolite that plays a central role in controlling ROS levels and maintaining cellular redox balance (Dorion et al., 2021). Increased glutathione production strengthens the antioxidant defense system, reduces oxidative damage, and enhances drought tolerance in soybean roots.

Drought stress leads to excessive ROS accumulation, which can cause serious damage to cellular components. Dehydrins (DHNs) and ferritins (FERs) are important protective proteins that help plants cope with oxidative stress. These proteins support water retention, protect chlorophyll, stabilize the photosynthetic machinery, activate ROS detoxification pathways, and promote the accumulation of compatible solutes (Ravet et al., 2009; Riyazuddin et al., 2022). In the soybean ‘Taegwang’, increased accumulation of dehydrins and ferritins in roots under drought stress has been linked to reduced oxidative damage (Alam et al., 2010). Differences in ferritin expression have also been observed between tolerant and sensitive soybean genotypes, possibly due to lower oxidative stress in tolerant, stay-green plants (Yahoueian et al., 2021). Ferritin accumulation was also reported in the pod wall under terminal drought stress (Sengupta et al., 2018). Dehydrins accumulate during soybean seed development, reaching maximum levels at seed maturity, and have been suggested as an indicator for seed maturation (Samarah et al., 2006). Higher dehydrin levels have consistently been observed in drought-tolerant soybean varieties compared with sensitive ones (Arumingtyas and Savitri, 2013). Salicylic acid (SA) also contributes to drought tolerance by strengthening antioxidant defenses. Pre-sowing application of SA in soybean increased the abundance of defense-related proteins, including glutathione S-transferase, ascorbate peroxidase, and peroxiredoxin, and supported energy production by enhancing ATP synthase expression (Sharma et al., 2018). SA also helped maintain sink strength and nitrogen use efficiency under drought conditions.

Drought stress can disrupt protein folding within the endoplasmic reticulum (ER), making ER chaperones important for stress tolerance. Calnexin is a well-known ER marker protein involved in the folding and quality control of newly synthesized glycoproteins (Bergeron et al., 1994). In soybean, calnexin levels increased under osmotic stress induced by polyethylene glycol (PEG) treatment (Nouri and Komatsu, 2010), but decreased under prolonged stress and under drought, salinity, cold, and ABA treatments, which was associated with reduced root growth (Nouri et al., 2012). Calnexin interacts with heat shock cognate proteins, suggesting a role as a molecular chaperone during stress. Supporting this role, heterologous expression of rice calnexin (OsCNX) in tobacco improved germination and survival under osmotic and dehydration stress (Sarwat and Naqvi, 2013). Another important ER chaperone in soybean is the binding protein BiP (soyBiPD). Overexpression of BiP in plants reduced wilting, delayed leaf senescence, and helped maintain water potential under drought stress (Valente et al., 2009). In soybean, BiP expression increases several-fold under drought and heat stress, and four BiP genes have been identified (Cascardo et al., 2001; Figueiredo et al., 1997). Overexpression of soyBiPD has been shown to enhance drought tolerance in *Nicotiana tabacum* (Alvim et al., 2001). The pod wall plays a critical role in regulating reserve accumulation during soybean seed development (Lindoo and Nooden, 1976; Lindoo and Noodén, 1977)). Proteomic analysis of KI-treated soybean variety ‘JS335’ revealed that many drought-responsive proteins in the pod wall are involved in signaling and regulatory processes (Sengupta et al., 2019). A summary of the proteomics studies revealing soybean responses to drought and osmotic stress can be found in **Supplementary Table 3**.

Proteomic studies provide valuable insights into how soybean responds to drought at the molecular level by revealing key proteins and pathways involved in stress tolerance, recovery, and yield stability. Understanding these protein-level changes is essential for identifying targets that can be used to develop more drought-tolerant soybean cultivars in the future.

### 3.5 Metabolomics in soybean drought tolerance

Understanding the metabolic shift that occurs under drought stress is critical for identifying the biochemical mechanisms underlying drought tolerance in soybeans. Metabolomics, which involves high-throughput profiling of small-molecule metabolites, has emerged as a powerful tool for identifying biomarkers and related biochemical pathways involved in abiotic stress responses, including drought tolerance (Arbona et al., 2013; Kumar et al., 2021). By capturing a snapshot of biochemical changes, metabolomics enables the identification of metabolites involved in osmotic regulation, antioxidant defense, energy metabolism, and secondary metabolic processes, all of which have been associated with drought tolerance in soybeans (Silvente et al., 2012; Das et al., 2017).

Technological advancements in high-throughput profiling, including gas chromatography-mass spectrometry (GC-MS), liquid chromatography-mass spectrometry (LC-MS), and nuclear magnetic resonance (NMR), have allowed researchers to analyze comprehensive metabolic shifts between drought-tolerant and -sensitive soybeans (Silvente et al., 2012; Nam et al., 2019; Wang et al., 2017, 2022b, 2024b; Fu et al., 2020; Vital et al., 2022). These studies have revealed significant changes in primary metabolites, including sugars, amino acids, organic acids, and fatty acids, as well as secondary metabolites such as flavonoids, phenylpropanoids, and phytohormones. A summary of metabolomics studies on soybean drought tolerance, including objectives, genotypes, and utilized techniques, is presented in **Supplementary Table 4**. Based on these studies, **Supplementary Table 5** highlights metabolites identified across at least two independent reports and proposes them as potential biomarkers for drought tolerance, categorized by biochemical class.

These studies highlight key metabolic adjustments in drought-tolerant soybean genotypes, including osmolyte accumulation, modulation of the tricarboxylic acid (TCA) cycle, lipid remodeling, secondary metabolite biosynthesis, and phytohormone regulation—processes that collectively contribute to drought resilience. Osmolytes such as alanine, proline, inositol, sorbitol, and mannitol help maintain osmotic balance by preserving cellular hydration and protecting macromolecules under drought stress (Ozturk et al., 2021). Comparative metabolomic analyses have revealed that drought-tolerant soybean genotypes (e.g. Heinong 44, Tongyu03611) exhibit elevated levels of these osmolytes in leaf tissues compared to drought-sensitive ones (e.g. Heinong 65, Huiman06116), emphasizing their functional relevance (Silvente et al., 2012; Nam et al., 2019; Wang et al., 2019, 2022b, 2024b; Fu et al., 2020; Vital et al., 2022). The TCA cycle is involved in regulating carbon and energy fluxes in plants under drought conditions (Fernie et al., 2004). Increased levels of TCA cycle intermediates, such as citric, fumaric, malic, and succinic acids, have been observed in drought-tolerant soybean genotypes (Wang et al., 2019; Fu et al., 2020). This suggests that TCA cycle modulation is closely linked to drought adaptation mechanisms via the adjustment of energy metabolism. These findings demonstrate that drought tolerance in soybeans is underpinned by coordinated metabolic regulation of osmotic balance and energy metabolism under water-limited conditions. However, it is crucial to recognize that metabolic reprogramming is highly tissue specific. While leaf metabolomics reflects the maintenance of photosynthetic machinery, root metabolic profiles often exhibit distinct strategies focused on water uptake and soil interaction. Therefore, future studies must elucidate these leaf- and root-specific metabolomic strategies to provide a comprehensive understanding of drought tolerance.

Lipid metabolism plays a crucial role in maintaining membrane integrity during drought by regulating membrane composition and activating lipid-based antioxidant mechanisms to mitigate oxidative damage caused by reactive oxygen species (ROS) (Golldack et al., 2014). In GC-MS based untargeted metabolomics studies, drought-tolerant soybean genotypes (Huinan6116 and Tongyu3611) have been shown to exhibit higher levels of fatty acids and their derivatives in leaf tissues, which may help maintain cell structure and membrane stability under water-limited conditions (Wang et al., 2019; Fu et al., 2020). Another study demonstrated similar results with increased fatty acid levels in a genetically engineered drought-resistant line (ABF3-overexpressing Kwangan), which highlights the function of lipids and lipid remodeling in drought-tolerant mechanisms (Nam et al., 2019). In addition, beyond the changes in fatty acid abundance, the degree of lipid unsaturation is a determining factor in drought tolerance (He and Ding, 2020); maintaining a higher ratio of unsaturated fatty acids is essential for preserving membrane fluidity and preventing phase transitions that lead to electrolyte leakage under severe dehydration. These results indicate that alterations in lipid metabolism may enhance drought tolerance in soybeans by stabilizing cellular membrane structures and supporting antioxidant defenses under drought conditions.

Beyond primary metabolism, the upregulation of secondary metabolites, especially flavonoids and phenylpropanoids, serves as a critical antioxidant defense system to mitigate ROS-induced damage (Jogawat et al., 2021). Increases in phenylpropanoid-derived flavonoids have been observed in drought-tolerant soybeans in multiple studies, as identified through untargeted metabolomic profiling of leaves from wild (e.g., Huinan6116, Tongyu6311) or genetically engineered (BiP-overexpressing line) soybean genotypes (Coutinho et al., 2019; Wang et al., 2019, 2022c; Fu et al., 2020), suggesting these compounds may serve as antioxidants to mitigate oxidative stress under water deficit. Similarly, upregulation of the flavonoid biosynthetic pathway was observed in drought-tolerant soybean genotype Heinong 44, with 1.6-fold increased production of kaempferol, a flavonoid related to antioxidant activity and stress adaptation (Wang et al., 2022c). These flavonoids may serve as promising markers of metabolites for drought resistance.

Phytohormones such as abscisic acid (ABA), salicylic acid (SA), and jasmonic acid (JA) are also involved in regulating drought stress at the molecular level (Bueno and Lopes, 2020). Under drought conditions, ABA accumulation induces stomatal closure and osmotic adjustment, thereby enhancing water-use efficiency (Silvente et al., 2012). JA and SA contribute to defense and stress signaling pathways by reducing oxidative damage and promoting cellular homeostasis. GC-MS and LC-MS-based metabolomics studies have revealed that drought-tolerant soybean genotypes such as Tongyu06311 and Huinan06116, and ABF3-overexpressing transgenic lines derived from Kwangan, exhibit higher levels of these phytohormones, including ABA, JA, and SA, under PEG-induced or field drought conditions. These findings support the function of plant hormone signaling in defense and adaptive responses to water limitation (Nam et al., 2019; Wang et al., 2019; Fu et al., 2020). A recent pathway-based metabolomics study utilizing PI 603535 and Benning-derived lines revealed that drought-tolerant genotypes employ a conservative metabolic strategy characterized by lower baseline metabolite levels and selective reprogramming of the TCA and shikimate pathways to optimize resource allocation under stress (Lee et al., 2026).

Although the application of multi-omics approaches in crop improvement is still emerging, it is increasingly being used in soybean drought research (Coutinho et al., 2019; Zhao et al., 2021; Zhang et al., 2023a). Integrative metabolomics-transcriptomics strategies have successfully uncovered core metabolic mechanisms, including the rewiring of amino acid and phenylpropanoid pathways, that support drought adaptation (Zhao et al., 2021; Zhang et al., 2023a). Metabolomics combined with proteomics has elucidated protein-level adaptations in soybean drought tolerance by observing upregulated photosynthesis-related proteins such as sedoheptulose-1,7-bisphosphatase, along with elevated levels of amino acids, including proline, isoleucine, tryptophan, valine, leucine, histidine, asparagine, and methionine, and variation in phytohormone levels in drought-tolerant soybeans (Coutinho et al., 2019).

In addition to multi-omics, emerging research in epigenomics and metagenomics provides promising directions for understanding drought resistance. Although these findings have not yet been conducted in soybeans, studies have shown that epigenetic modifications such as DNA methylation and histone deacetylation regulate hormonal signaling pathways involved in drought responses across plants (Shaik and Ramakrishna, 2012; Kaya et al., 2024). The root-associated microbiomes, such as plant growth-promoting rhizobacteria and mycorrhizal fungi, have been shown to enhance drought tolerance in crops like rice and wheat by modulating phytohormone levels and other stress-related signaling pathways (Mathur and Roy, 2021; Trivedi et al., 2022). These insights suggest that incorporating epigenomics and microbiome analysis is expected to provide a deeper understanding of soybean drought resistance in the future. Despite these technological advances, a major bottleneck remains in distinguishing between adaptive accumulation (active tolerance) and stress-induced degradation products (passive injury) (Alseekh and Fernie, 2018; Widodo et al., 2009). Future research should therefore prioritize functional validation, e.g., exogenous metabolite application or metabolic engineering, to confirm the causal role of these potential biomarkers in conferring drought resilience.

### 3.6 Application of remote sensing technologies for drought tolerance phenotyping

Identifying drought-tolerant soybean genotypes is challenging due to the numerous physiological and morphological traits—both major and minor—that influence drought response (Purcell and Specht, 2004). Physiological traits such as stomatal conductance, leaf carbon isotope discrimination [CID, a proxy for water-use efficiency (WUE)], and nitrogen fixation require time-sensitive tissue collection or in-field measurements that are often labor-intensive (Ries et al., 2012; Steketee et al., 2020). Morphological traits such as root architecture or whole-plant CID require destructive and labor-intensive phenotyping (Abdel-Haleem et al., 2011; Costa Netto et al., 2025). CW ratings are non-destructive and less labor-intensive than phenotyping for primary drought traits; however, they are still time-consuming, subjective, and prone to human error and bias (Bock et al., 2010).

Unmanned aerial vehicles (UAVs) have become a widely adopted method for high-throughput phenotyping (HTP) in plant breeding, due to their ability to capture imagery across thousands of plots within minutes (Araus et al., 2018; Sankaran et al., 2015; Liang et al., 2024; Zhang et al., 2023b; Kim and Lee, 2025). In soybean, UAV-based remote sensing has been used to phenotype symptoms of biotic and abiotic stress, including nutrient deficiencies, flooding, herbicide injury, nematode infestation, and foliar diseases (Burner et al., 2025; Burner, 2025; Castelão Tetila et al., 2017; Dobbels and Lorenz, 2019; Santos et al., 2022; Vieira et al., 2022; Zhou et al., 2021). Remote sensing has only recently been used to phenotype soybean drought stress in the field. Bai and Purcell (2018) used aerial thermal imagery to measure canopy temperature (CT) of 10 soybean lines and found that CT generally increased with greater drought stress. Additionally, slow CW lines tended to exhibit lower CT compared to fast CW lines under drought stress. These differences were observed even in the absence of differences in wilting phenotyping, indicating that CT may be a useful trait for screening for drought tolerance. The lower CT under drought stress was attributed to higher stomatal conductance in slow CW lines compared to fast CW lines, resulting in cooler canopies due to transpiration, compared to fast CW lines (Bai and Purcell, 2018; Jones et al., 2009). The authors hypothesized that slow CW lines access more water during drought periods. Supporting this, Zhou et al. (2020) found that slow CW lines could be distinguished from fast CW lines using CT in a larger set of 103 genotypes. Additionally, slow CW lines exhibited significantly higher Normalized Difference Vegetation Index (NDVI), hue, and saturation values. A support vector machine was developed using these traits, as well as other remote sensing traits, NDVI, Green Normalized Difference Vegetation Index (GNDVI), CT, hue, and saturation, canopy size and height, exhibiting an accuracy of 80% for classifying lines as slow or fast wilting.

CT has been used as a phenotype for mapping studies to identify genomic regions responsible for drought-related traits such as CW, WUE, root morphology, canopy coverage, carbon isotope ratio, and stress signal transduction (Burner et al., 2025; Burner, 2025; Bazzer and Purcell, 2020; Kaler et al., 2018). Kaler et al. (2018) identified 34 and 8 loci associated with CT in single- and combined-year genome-wide association mapping (GWAM) of 345 MG IV accessions. Fifteen of the loci were located within previously annotated genes, and 15 loci overlapped with those previously associated with CW (Kaler et al., 2017). Additionally, CT and CW exhibited a significant positive correlation (R = 0.25) when averaged across environments.

Bazzer and Purcell (2020) identified 11 loci associated with CT on eight different chromosomes through QTL analyses in both single and across-environments of a KS4895 × Jackson RIL population. All identified loci co-localized with previous QTLs associated with the aforementioned drought traits. Notably, Chr 11 QTL explained the highest proportion of CT variation across environments, which coincided with a region associated with the carbon isotope ratio previously identified in the same population (Bazzer and Purcell, 2020). Burner et al. (2025) used NDVI, GNDVI, NDRE, and CT as additional phenotypes for mapping genomic regions associated with drought tolerance in a Benning × PI 603535 RIL population. The multispectral indices NDVI and GNDVI consistently exhibited strong correlations with the CW score, both across and within years. In contrast, CT was weakly correlated with CW scores, likely due to the greater influence of environmental conditions on the trait compared to other indices. The two largest CW QTLs by percent variation explained identified in the study co-localized with QTLs for GNDVI and NDVI, indicating potential utility for identifying underlying genetic variation associated with drought tolerance.

After evaluating soybean genotypes under drought stress using multimodal UAV data and machine learning, Liang et al. (2024) indicated that the accuracy of UAV-based predictions of drought injury scores gradually increased as soybean growth progressed, reaching a maximum of 77.1% at maturity. Similarly, Zhang et al. (2023b) developed an accurate and rapid drought evaluation method based on feature extraction of multispectral images from soybean canopy which reached 96.9% of accuracy. Using bio-speckle and hyperspectral imaging, Kim and Lee (2025) imaged soybean plants under four water treatment levels and observed significant difference among the treatments, indicating this multimodal system could be used effectively to phenotype drought tolerance. These findings support the utility of remote sensing traits, providing a more standardized and objective HTP method for identifying soybean lines with improved drought tolerance.

Additionally, traits such as CT have been used to identify genomic regions associated with drought-related traits that are traditionally tedious to phenotype. Although there are substantially fewer studies assessing CT in soybean, evidence so far indicates that it is more influenced by the environment than CW. Light-based indices such as NDVI and GNDVI appear to have greater utility in consistently evaluating relative drought tolerance due to their association with chlorophyll content and photosynthetic rate (Candiago et al., 2015; Gitelson et al., 1996). Zhou et al. (2020) found that NDVI and GNDVI exhibited heritabilities of 67 and 81%, respectively, although GNDVI was not found to be significantly different between slow and fast CW lines. Consequently, researchers must weigh the trade-offs between trait heritability and phenotyping efficiency when incorporating remote sensing data into genetic studies.

With advances in remote sensing technologies, solar-induced chlorophyll fluorescence (SIF) has emerged as a useful tool for studying plant physiological responses to drought stress. SIF is a weak light signal emitted by chlorophyll when plants absorb sunlight during photosynthesis (Behera et al., 2025). Compared with traditional vegetation indices such as NDVI, GNDVI, and enhanced vegetation index (EVI), SIF offers a distinct advantage for direct and rapid detection of plant stress responses at leaf, canopy, UAV, and satellite scales (Xu et al., 2023a; Wu et al., 2024; Wang et al., 2022a).

To date, most applications of SIF have focused on predicting gross primary productivity (GPP), defined as the total amount of CO_2_ fixed by plants during photosynthesis (Mamić et al., 2025; Wang et al., 2022a). Much of this research has focused on meteorological drought at regional to global production scales (Wu et al., 2024). For example, using crop production data across India, Behera et al. (2025) demonstrated that SIF exhibited the strongest positive correlation with vegetation condition and proposed SIF as an immediate physiological indicator of drought stress. Similarly, Liu et al. (2022) reported that SIF responds rapidly to drought events, showing a significant decrease than NDVI, EVI, and near-infrared reflectance of vegetation (NIRv) across vegetation types. Relatively few studies have examined SIF responses in soybean. Investigating the effects of elevated ozone on canopy structure and senescence, Wu et al. (2024) found that soybean plants that exposed to elevated O_3_ exhibited a significant reduction in canopy-level SIF. Both SIF and GPP are influenced by crop type, canopy architecture, and environmental conditions. Consequently, the relationship between SIF and GPP is not always linear.

### 3.7 Breeding for enhanced drought tolerance

Historically, soybean breeding programs in the United States have not prioritized the development of cultivars explicitly selected for improved drought tolerance. Instead, breeding efforts largely focused on minimizing G × E interactions for important agronomic traits (Schutz and Bernard, 1967). Drought conditions tend to be unpredictable in terms of frequency and duration from year to year, presenting a challenge to breeders. Many traits that mitigate drought losses under stressed conditions tend to underperform compared to more sensitive cultivars under favorable conditions (Blum, 2017; Richards, 1996). Therefore, the economic advantage of a tolerant cultivar during stressed years must significantly outweigh potential yield penalties in favorable years (Purcell and Specht, 2004).

Despite the absence of explicit soybean breeding efforts targeting drought tolerance, there is evidence that selecting higher-yielding cultivars has resulted in the indirect selection of drought avoidance traits, such as increased root density and reduced midday leaf water deficits (Boyer et al., 1980). The first soybean cultivar identified to exhibit reduced drought sensitivity was found in an evaluation of yield changes under differential irrigation levels (Specht et al., 1986). The authors reported no significant correlation between a cultivar’s yield response to irrigation treatments and its grand mean yield across those treatments (Specht et al., 1986). These results highlight breeders’ preference for genotypes with broad adaptation over those with specific adaptation. The results of Specht et al. (1986) were consistent with previous observations of a general lack of significant cultivar × moisture stress interactions among commercial soybean cultivars (Brown et al., 1985). At the time of these studies’ publication, no U.S. commercial soybean cultivars had been released specifically for drought tolerance (Sloane et al., 1990). More recent reviews, however, confirm that these foundational insights still hold, as efforts to breed for broad adaptability continue to dominate soybean improvement programs in the face of environmental variability (Arya et al., 2021).

Carter (1999) proposed several barriers that have prevented the development of elite drought-tolerant soybean cultivars. First is skepticism among breeders about whether drought tolerance traits would harm yield performance in normal or wet years, non-stressed environments. Additionally, yield trials are often conducted under well-watered conditions to better assess yield potential, which can lead to the exclusion of data from low-yielding locations. Moreover, there is a lack of genetic variability for drought tolerance among modern soybean cultivars, likely due to the narrow genetic base from which most are derived (Brown et al., 1985; Carter et al., 2004; Gizlice et al., 1994; Sneller and Dombek, 1997). Third, increased soil heterogeneity under drought conditions reduces precision in evaluating soybean lines (Carter, 1999). Lastly, drought studies are inherently time-consuming and resource-intensive due to the unpredictability of seasonal drought conditions, often resulting in multiple years of poor data (Carter, 1999). Furthermore, the timing and intensity of drought conditions vary significantly from year to year, which hinders the reproducibility of screenings (Specht and Williams, 1985).

Nevertheless, progress has been made through the identification of soybean lines exhibiting drought tolerance with the slow CW phenotype. PI 416937 (Japan) and PI 471938 (Nepal) have been widely used in breeding programs to develop cultivars with improved drought tolerance, particularly in the southeastern United States (Devi et al., 2014; Grant et al., 2010). Chinese accessions PI 567690 and PI 597731 (MG III) were the first identified slow CW lines within maturity groups common to the U.S. Midwest (Pathan et al., 2014). Recently, Burner et al. (2025) identified PI 603535 (MG VIII) as a slow CW line. These findings underscore the value of exotic germplasm in breeding programs designed to enhance drought resilience.

Building upon these identified traits, public breeding programs have released several drought-tolerant germplasm lines and cultivars. USDA-N8002 was released in 2016 as an MG VIII conventional cultivar by the USDA-ARS (Carter et al., 2016). Derived from a cross between N7002 and N98-7265, N8002 includes 25% of its pedigree from PI 471938 and 12.5% from PI 416937. It was the first release derived from PI 471938 and the second from PI 416937. N8002 exhibits slow canopy-wilting, sustained N fixation under drought stress, and water-conserving transpiration in response to a vapor pressure deficit. In 74 environments across the Southeastern U.S., N8002 outyielded the check cultivar N8001 by more than 5%, highlighting its high yield potential (Carter et al., 2016). In 2020, two drought-tolerant MG V conventional lines, R10–2436 and R10-2710, were released by the Arkansas Agricultural Experiment Station (Manjarrez-Sandoval et al., 2020). R10–2710 originated from a cross between R01-52F × N7002 and derived 25% of its pedigree from PI 416937 and exhibits slow wilting. R10–2436 was derived from a cross between R01-52F and R02-6268F. Both lines exhibit sustained nitrogen fixation under drought stress, likely tracing back to Jackson. Released in 2023, USDA-N7006 is an MG VII conventional line developed from TCPR01-83 × N01-1136 (Fallen et al., 2023). It traces 12.5% of its pedigree to PI 416937 and 25% to PI 407859-2, making it the first North American release incorporating PI 407859–2 from South Korea. In drought-stressed trials across North and South Carolina, N7006 exhibited less canopy wilting and more stable yields than N8002, further advancing breeding efforts to improve drought tolerance in soybean. TN16-5201R1, released by the University of Tennessee in 2024, was developed from the recurrent parent Ellis and the glyphosate-tolerant donor parent TN13-4730R1 (Smallwood et al., 2024). TN13-4730R1 and Ellis have been shown to have high yield and slow canopy wilting under drought stress (Purdom et al., 2022). TN16-520R1 has also been shown to have a stable protein content and consistent performance under dryland and irrigated field trials across the Mid-South, similar to Ellis. The most recent release, R19-42848, was released in 2025. R19–42848 was derived from breeding lines R12-2237 × R12-519 (Wu et al., 2025). Its pedigree includes R01-52F, Jackson, and PI 416937. R19–42848 demonstrated high yield and broad adaptability across multiple years and environments. Under dryland field conditions, it maintained strong performance with slow canopy wilting and yielded statistically similar to Ellis.

In addition to these releases, ongoing research has identified other germplasm lines with traits favorable for drought tolerance. NTCPR94–5157 has been recognized for its ability to penetrate compacted soil layers, a trait associated with improved water uptake under drought conditions. NTCPR94–5157 was the only genotype reported to fully penetrate the hardpan (Fried et al., 2018). These ongoing advancements underscore the importance of incorporating diverse genetic resources into soybean breeding programs to enhance drought resilience. The utilization of exotic germplasm sources has proven effective in introducing beneficial traits, and continued exploration and integration of such resources are vital for developing cultivars capable of withstanding the challenges posed by abiotic stress.

## 4 Discussion and prospect

### 4.1 Physiological and root-based mechanisms contributing to slow canopy wilting under drought stress

Recent work has shown that CW integrates whole-plant hydraulics with photosynthetic efficiency, where drought-induced stomatal closure leads to reduced CO_2_ assimilation, oxidative stress, and changes in photosystem II activity, all of which can be detected through chlorophyll fluorescence parameters as demonstrated during progressive water-deficit treatments in soybean (Falcioni et al., 2025). Root traits provide another critical layer of control over CW expression. Genotypes with deeper and more branched root systems maintain access to subsoil water reserves, thereby sustaining transpiration and delaying loss of leaf turgor. Recent physiological analysis demonstrated that drought-resistant soybean varieties accumulate higher levels of flavonoids and phenolic acids in roots, maintain stronger antioxidant enzyme activity, and upregulate genes in the phenylpropanoid and isoflavonoid biosynthesis pathways, all of which contribute to improved water uptake and drought resistance (Wang et al., 2024c). These findings emphasize that CW is not solely a leaf-level trait but rather reflects the integration of below and aboveground processes, including adjustments in the root system that safeguard canopy function under drought.

### 4.2 Leveraging multi-omics technologies for understanding and selection of drought tolerance

While previous research using transcriptomics to study drought response in soybeans has uncovered many genes and pathways involved in drought tolerance, most of this research was conducted in either a laboratory or greenhouse setting, with a few in a field setting. However, it has been reported that the differential expression to drought stress is significantly different between the greenhouse or laboratory and the field (Lovell et al., 2016). Therefore, to understand soybean drought tolerance in field conditions, there is a need to evaluate hypotheses in realistic environmental conditions. Doing this will contribute to our understanding of how genetic mechanisms function under complex environmental conditions. Further, because the field conditions expose soybeans to abiotic stresses, with some interacting, there is a need to understand how soybean responds to such interactions of abiotic stresses at the transcriptomic level (Pandey et al., 2015). This will aid efforts in the development of multi-stress resilient soybean varieties. Many studies conducted in the past have primarily focused on the seedling stage, assaying leaf samples, while a few studies have focused on other tissues such as sepals, stigma, roots, and pods. To gain a comprehensive system-level understanding of soybean drought tolerance, it is imperative to conduct more studies on the temporal and spatial changes in the transcriptome of soybeans in response to drought (Xia et al., 2020). This can be achieved by using time series data collection methods that focus on different tissues at different developmental stages.

The advances in recent years, such as the advent of artificial intelligence and machine learning, and multi-omics methods, have led to new applications for transcriptomics in soybean. Integrating artificial intelligence and machine learning into the analysis of the huge transcriptomic datasets can yield a deeper understanding of soybean drought tolerance. Machine learning algorithms are great at finding patterns. Therefore, their use can bring to light hidden gene regulatory networks and predict the function of genes under drought stress, and by extension, other abiotic stresses (Panahi et al., 2025). Further, the use of different omics methods, such as proteomics, metabolomics, and phenomics combined with transcriptomics, presents the opportunity to gain a holistic understanding of drought tolerance in soybean. Researchers can use such holistic studies to develop models and gene networks to identify hub genes and find out which genes influence important traits such as canopy wilting (Ahmed and Kabir, 2022).

Most proteomics studies on soybean drought tolerance have concentrated on the seedling stage, with limited research on reproductive stages. However, to understand the temporal and spatial changes in the proteome, studies should include time points that cover the onset, peak, and recovery from drought stress, as well as focus on different tissues at various developmental stages (Hossain and Komatsu, 2014). Additionally, proteomics studies examining combined abiotic stress responses are underrepresented, and the integration of proteomics and transcriptomics for drought stress in soybean is very limited. However, with many omics approaches available, combining proteomics with other omics such as transcriptomics, metabolomics, and phenomics offers deeper insights into the system-level drought response in soybean (Deshmukh et al., 2014).

Metabolomics research has provided valuable insights into soybean drought tolerance, yet several aspects of the adaptive processes remain incompletely understood. Many studies emphasize general trends in metabolite accumulation but offer limited understanding of how these responses are influenced by genotype or connected to physiological traits relevant to drought tolerance. Since current approaches are often not designed to identify metabolites associated with measurable phenotypic variation, future research should apply more multi-omics strategies combined with phenotypic assessments to identify key regulatory components involved in metabolic adjustment. High-throughput phenotyping and machine learning approaches can further enhance biomarker discovery by linking metabolic profiles and networks to drought resilience traits.

### 4.3 Utilizing remote sensing technologies to enhance selection for drought tolerance

Drought tolerance is a complex trait in soybean (Menke et al., 2024; Burner et al., 2025). It is influenced by genotype, environment, and their interactions. The response of plants to drought stress varies across plant growth stages and environments. Due to the complexity and environmental influence on drought response, selecting drought-tolerant soybean genotypes is challenging (Purcell and Specht, 2004). Traditionally, phenotyping for drought tolerance such as canopy wilting and rooting is often time consuming and would not be able to capture all aspects of drought tolerance. Remote sensing implemented by unmanned aerial vehicles (UAVs) has become a widely adopted method for phenotyping plants for various traits such as biotic and abiotic stress, nutrient deficiencies, flooding, herbicide injury, nematode infestation, and foliar diseases in multiple crops (Burner et al., 2025; Burner, 2025; Castelão Tetila et al., 2017; Dobbels and Lorenz, 2019; Santos et al., 2022; Vieira et al., 2022; Zhou et al., 2021; Balota and Oakes, 2017; Li et al., 2022b; and Das et al., 2022). It has been used to phenotype soybean drought stress in the field on a large scale (Bai and Purcell, 2018; Jones et al., 2009; Zhou et al., 2020; Zhang et al., 2023b; Liang et al., 2024; Kim and Lee, 2025; Burner et al., 2025). Of these remote sensing traits, NDVI, GNDVI, thermal imagery, and photochemical reflectance index (PRI) have been used in drought phenotyping. NDVI, GNDVI, and PRI typically measure canopy greenness and photosynthetic efficiency, while thermal imagery functions as a proxy for stomatal conductance and transpiration rate of plants.

These results support the utility of remote sensing traits, providing a more standardized and objective HTP method for identifying soybean lines with improved drought tolerance. Additionally, these methods have also been used to identify genomic regions associated with drought-related traits that are traditionally tedious to phenotype. Recent advances in remote sensing have enabled the use of SIF to directly estimate photosynthetic activities and plant responses to drought stress. However, most established relationships between SIF and GPP have been derived from large-scale, global assessments of crop production. In contrast, plant breeding for drought tolerance requires high-throughput evaluation of tens of thousands of individual genotypes or plots to identify and select drought-tolerant soybean genotypes. Therefore, future research should prioritize experiments at the plant and plot canopy scales to improve the prediction of photosynthetic performance under drought stress using SIF (Helm et al., 2020; Pandiyan et al., 2022). Additional studies are needed to bridge the gap between global-scale SIF applications and breeding-scale phenotyping.

Although remote sensing offers a promising toolkit for modern breeding programs to discover QTLs and develop drought-tolerant cultivars. However, challenges remain in data acquisition, standardization, data analysis, and interpretation. Data analysis for these traits requires researchers with specialized skills and is time-consuming. It is also challenging to link remote sensing traits to physiological mechanisms. Continued optimization of the remote sensing process to make it a more dependable tool will be essential for drought-stress phenotyping.

### 4.4 Integrating genomic prediction with remote sensing to predict and select for drought tolerant plants

Genetic studies and QTL mapping suggested that CW and other drought-tolerance related traits are complex and influenced by multiple genetic mechanisms, often acting in tandem or independently. Moreover, the effects of these QTLs identified are often small. The utility of identified genomic regions for breeding drought tolerance through marker-assisted selection may be limited by the magnitude of QTL effects and variable stability across environments. Therefore, extensive testing and confirmation of these QTLs are required to effectively incorporate favorable QTL alleles into a drought tolerance improvement scheme.

Genomic prediction has become a useful tool in plant breeding. It leverages genome-wide marker data and their associations with plant phenotypes to estimate breeding values for complex traits such as yield without requiring QTL information. The procedure has been widely used in multiple crops for different traits. However, the power of GP relies on many factors including quality of phenotypic data and relatedness of training set to the testing materials.

**Figure 1:**
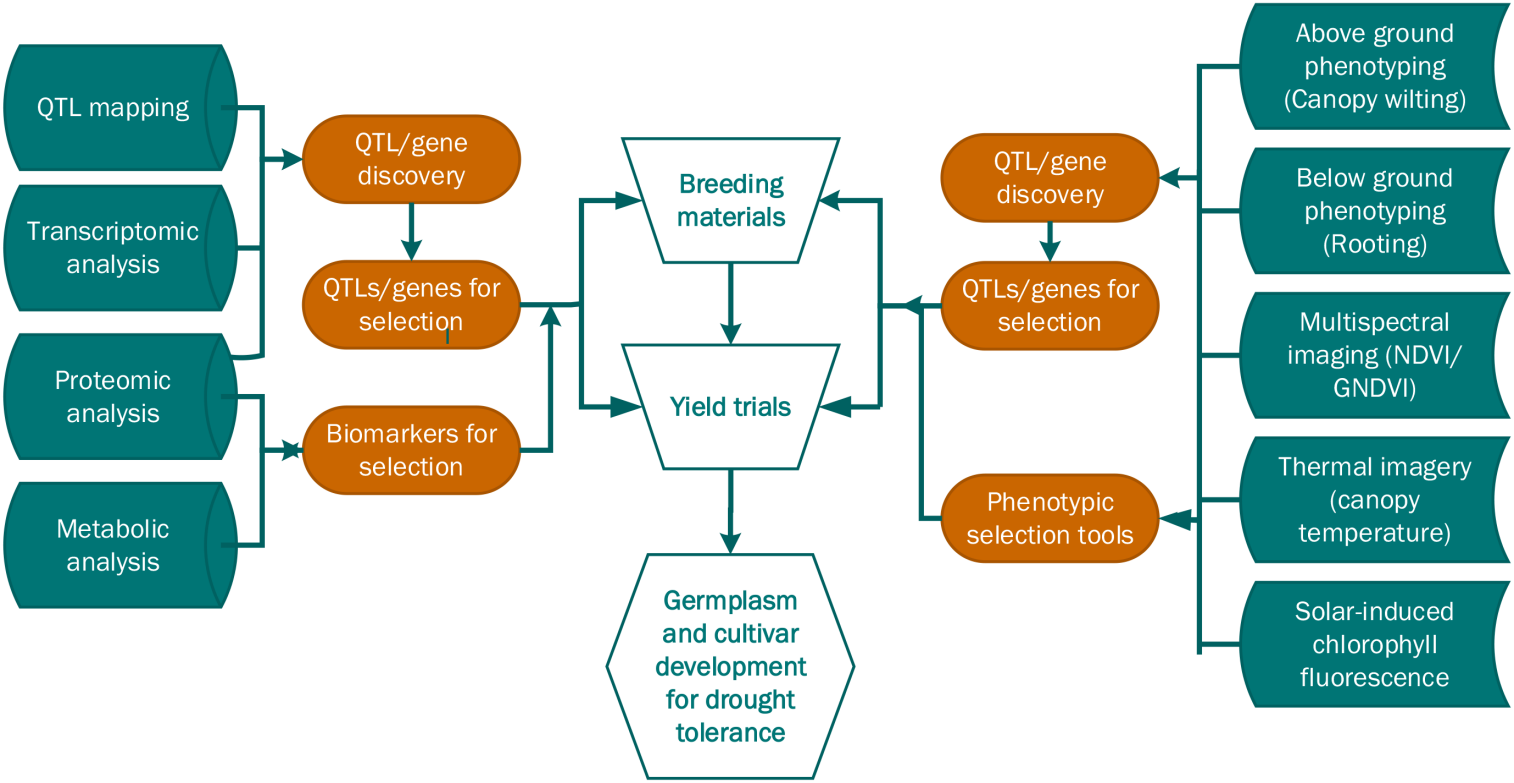
Utilizing omics technologies for selection of drought tolerance.

Remote sensing technologies, such as multispectral, hyperspectral, and thermal imaging, have enabled high-throughput, non-destructive phenotyping, which provides a dynamic view of plant performance under drought stress. These remote sensing traits can be included in GP models for prediction. Therefore, integrating GP with remote sensing traits will offer a powerful approach to enhance selection accuracy and efficiency for drought tolerance breeding to develop drought-tolerant cultivars. A diagram that integrated omics technologies for selection of drought tolerance in a breeding program is presented in **Figure 1**.

Despite these advancements, the continued efforts should focus on optimizing both GS and remote sensing processes to develop robust multi-trait models and to streamline the process to efficiently integrate the data and perform data analysis. In addition, artificial intelligence models such as machine learning and deep learning algorithms can be used to analyze genomic and remote sensing data to dissect genotype-by-environment interactions which then provide a reliable method for selection of drought tolerance in plant breeding.

### 4.5 Future roadmap for drought tolerance soybean breeding

Drought tolerance in soybean is a complex, multigenic trait, and continued progress will depend on adopting a system-based approach to phenotyping and selection that integrates genetics, physiology, and field performance. To accelerate the development of drought-tolerant cultivars capable of maintaining stable performance under increasingly variable water-limiting conditions, future research should prioritize coordinated, field-relevant approaches. Key priorities include: 1) precise phenotyping of CW and root system architecture using advanced field-based technologies such as remote sensing, coupled with a deeper understanding of the relationship among CW, root traits, soil water dynamics and yield under rain-fed or managed drought conditions; 2) elucidating the relationship between drought responses expressed at the seedling and adult plant stages to improve selection consistency across development stages; and 3) integrating multi-omics approaches, including QTL mapping, transcriptomics, proteomics, and metabolomics, with genomic prediction and artificial intelligence frameworks to move beyond single-gene markers and uncover the regulatory networks and metabolic processes that underlie drought tolerance at the system level. Collectively, these efforts are expected to improve the breeders’ ability to predict genotype performance under drought stress conditions and accelerate genetic improvement for drought tolerance while maintaining agronomic performance.

## Supplementary material

Supplementary Table 1 Summary of QTLs identified for drought tolerance using different mapping methods in soybean. MG, maturity group; QTL, quantitative trait locus; QTN, quantitative trait nucleotide; GWAS, Genome-wide association study; Chr, Chromosome.

Supplementary Table 2 Summary of transcriptomic studies on soybean responses to drought stress conditions.

Supplementary Table 3 Summary of proteomics studies revealing soybean responses to drought and osmotic stress.

Supplementary Table 4 A summary of the literature on the application of metabolomics in soybean drought tolerance research.^*^ GC-MS, gas chromatography/mass spectrometry; NMR, nuclear magnetic resonance; LC-MS, liquid chromatography-mass spectrometry; LC-QTOF-MS, liquid chromatography-quadrupole time of flight-mass spectrometry; GC-TOF-MS, gas chromatography-time of flight-mass spectrometry; 2DE-nanoLC-MS, two-dimensional gel electrophoresis-nanoflow liquid chromatography-mass spectrometry; RNA-Seq, RNA sequencing.

Supplementary Table 5 List of potential metabolite markers selected from soybean drought tolerance research (Silvente et al., 2012; Coutinho et al., 2019; Nam et al., 2019; Wang et al., 2019, 2022b, 2024b; Fu et al., 2020; Zhao et al., 2021; Vital et al., 2022; Zhang et al., 2023).
