# Supplementary material for: Drought tolerance in soybean: genetics, metabolomics, remote sensing, and breeding for enhanced drought tolerance
Source: Front Plant Sci. 2026 Mar 13;17:1733525. doi: 10.3389/fpls.2026.1733525 (PMC13021458; doi:10.3389/fpls.2026.1733525)
Supplement: Supplementary Table 1 — Summary of QTLs identified for drought tolerance using different mapping methods in soybean. MG, maturity group; QTL, quantitative trait locus; QTN, quantitative trait nucleotide; GWAS, Genome-wide association study; Chr, Chromosome. [file DataSheet1.docx]

**Supplemental Table 1.** Summary of QTLs identified for drought tolerance using different mapping methods in soybean

| **Population / Panel** | **Method** | **QTLs / Loci Identified (Chromosomes)** | **Major Effects / Key Findings** | **Reference** |
| --- | --- | --- | --- | --- |
| KS4895 × Jackson  RILs | QTL mapping  Canopy wilting | Chrs 8, 13, 14, & 17 | Chr 17 explained 15% of phenotypic variance; Chr 13 detected across environments | Charlson et al. (2009) |
| Kefeng1 × Nannong1138-2 RILs | QTL mapping  Canopy wilting | 8 QTLs | Chr 8 QTL explained 24% of phenotypic variance: two QTL stable across field and greenhouse | Du et al. (2009) |
| Benning × PI 416937  RILs | QTL mapping (fibrous roots) | Chrs 1, 3, 4, 8, & 20 | Each explained 7.3–13.5% variance; only Chr 4 co-localized with CW QTL | Abdel-Haleem et al. (2011) |
| Benning × PI 416937  RILs | QTL mapping  Canopy wilting | Chrs 2, 4, 5, 12, 14, 17, & 19 | QTLs explained 75% total variance: Chr 12 largest effect (27%) | Abdel-Haleem et al. (2012) |
| 5 RIL populations | Multi-population QTL analysis  Canopy wilting | Chrs 2, 5, 11, 17, & 19 | Only Chr 17 and Chrs 2 & 5 consistently detected | Hwang et al. (2015) |
| Meta-QTL analysis | Meta-analysis  Canopy wilting | Meta-QTLs on Chrs 2, 5, & 17 | Six stable meta-QTLs useful for MAS; Chr 11 and 19 major but unstable | Hwang et al. (2016) |
| 373 MG IV accessions | GWAS  Canopy wilting | 51 loci (including Chrs 2, 6, 11, 17, 18, &19) | Strongest SNP on Chr 18; Chr 6 linked to ABA response; overlaps with known QTL clusters | Kaler et al. (2017) |
| 162 MG VI–VIII accessions | GWAS  Canopy wilting | 44 SNPs on 19 chromosomes | Major effect QTL on Chr 2 | Steketee et al. (2020) |
| Pana × PI 567690 & Magellan × PI 567731 RILs | QTL mapping  Canopy wilting | Chrs 2, 4, 5, 9, 12, 17, &19; Chrs 6 &10 | Chr 6 explained up to 29.6% | Ye et al. (2020) |
| Benning × PI 471938 RILs | QTL mapping  Canopy wilting | Chrs 2, 8, & 9 | Each explained 10–14% variance; aligned with prior QTLs | Menke et al. (2024) |
| Benning × PI 603535 RILs | QTL mapping  Canopy wilting | Chrs 2 (2), 3, 7, 12, 13, & 19 | Chr 12 largest effect (10.3%) | Burner et al. (2025) |
| 200 MG IV accessions | GWAS  Canopy wilting | 188 significant SNPs associated with 152 loci | 87 SNPs associated with 68 loci overlapped with previous report | Chamarthi et al., 2021 |
| 240 accessions | GWAS  Seedling survival ability at V2 stage | 23 & 27 QTNs were found in two models, respectively | 3 QTNs identified with two models and a QTN on Chr 8 was associated with two traits | Aleem et al. (2024) |
| 207 diverse accessions | GWAS  Seed germination rate | 58 QTLs were detected | 10 QTLs explained a larger portion of phenotypic variation | Kong et al. (2025) |
| Zhonghuang 35 × Jindou 21 RILs  259 accessions | QTL mapping and GWAS  Seedling survival ability at V2 stage | 9 QTL clusters from QTL mapping and 53 QTLs from GWAS | Two QTLs were in common across two populations | Zhang et al. (2022) |

Note: MG = maturity group; QTL=quantitative trait locus; QTN=quantitative trait nucleotide; GWAS = Genome-wide association study; Chr = Chromosome

**Supplemental Table 2.** Summary of transcriptomic studies on soybean responses to drought stress conditions.

| **Genotype/ varieties** | **Tissue** | **Stage** | **Stress type, level and duration** | **Key findings** | **Major Pathways/Genes** | **Reference** |
| --- | --- | --- | --- | --- | --- | --- |
| DT – (PI 416937) and DS – (Benning) | Leaf | R2 | Plants were uprooted, roots washed and whole plant exposed to air for 0, 6, 12, and 24 hrs. for water deficit | Downregulation of photosynthesis and the upregulation of protein transport and chromatin remodeling in response to water deficit | Photosynthesis, protein transport, and chromatin remodeling | Shin et al., 2015 |
| William 82 | Root | V3 (BBCH: 12) | Withholding water for 5 d very mild, 12 d for mild, 19 d for severe stress, and water recovery where plants were rewatered for 2 d after severe water stress | Among the alternative splicing events identified, the major types were alternative 3’ splice sites and skipped exons | Splicing regulatory factors in the spliceosome pathway and mRNA surveillance pathway | Song et al., 2020 |
| C03-3 wild-type and transgenic lines (with repressor form of *GmbZIP15)* | Leaf | 2 weeks | Salt (200 mM NaCl) and drought (300 mM mannitol) stress for 24 h | *GmbZIP15* positively regulates *GmSAHH1* expression and negatively regulates *GmWRKY12* and *GmABF1* expression in response to both salt and drought stress conditions | *GmSAHH1, GmWRKY12, GmABF1* | Zhang et al., 2020 |
| DT – (PI 342618B and A214) and DS – (NN86-4 and A195) | Root | 4 days of germination | 15% polyethylene glycol (PEG) - 6000 | Eight of the identified genes were located on Chromosome 8 in the regions previously identified to be hotspots associated with drought tolerance in soybean | Water and auxin transport, cell wall/membrane, antioxidant activity, catalytic activity, secondary metabolism, signaling and transcription factor activities. | Aleem et al., 2021 |
| DS – (BR16) | Flowers and pods | R2 and R4 | Withholding water for 7 days | DEGs in flowers were downregulated, but during pod fill, the DEGs were upregulated | Transition metal ion binding, transferase activity, ion binding, and hydrolase activity | Correa Molinari et al., 2021 |
| DS – (Liaodou 15) | Leaf | Early flowering stage | Withholding irrigation for 7 d for mild, 17 d for moderate, and 27 d for severe stress) and a control in field conditions | The soybean plant is able to activate the genes of antioxidants, secondary metabolism and hormone signaling pathway in response to drought stress | ABA, flavonoid biosynthesis, ascorbic acid, and glutathione metabolism | Li et al., 2022a |
| DT – (Heinong 65) and DS – (Heinong 44) | Leaf | V3 (BBCH: 12) | 15% PEG-6000 for four days | Drought alters the biosynthesis and signal transduction of other plant hormones, including auxin, gibberellin, brassinolide, and abscisic acid, and promotes substantial changes (increase or decrease) in flavonoids. | Tricarboxylic acid cycle (TCA) cycle and isoflavone biosynthesis pathway | Wang et al., 2022c |
| DT – (Embrapa 48) and DS – (BR16) | Leaf and root | V3 (BBCH: 12) | Exposing roots to air for 0 min for control, 25 – 50 min for mild, 75 – 100 min for moderate, and 125-150 min for severe water deficit | Root tissue showed more ABA-responsive genes and pathways triggered in response to water deficit conditions in both the drought- tolerant and -susceptible cultivars | Abscisic acid (ABA)-response pathway | Molinari et al., 2023 |
| DT - Heinong 44 (HN44) and DS -Suinong 14 (SN14) | Leaf | R5 | Normal water (NW) - 70% pot soil water capacity (PSWC), light drought (LD; 60%PSWC), moderate drought (MD; 55%PSWC), and severe drought (SD; 50%PSWC), | Expression of genes encoding proteins in photosynthesis pathway was inhibited and genes regulating in glutathione metabolism were upregulated which gives stronger ability to clear ROS. | Photosynthesis pathway, glutathione metabolism, MAPK signaling pathway and plant hormone signal transduction | Xu et al., 2023b |
| DT – (SS2-2) and DS –(Taekwang) | Leaf | V3 (BBCH: 12) | Water was withheld for 8 days | Differentially expressed genes (DEGs) were involved in lipid metabolism pathways in the drought-tolerant cultivar; however, in the drought-susceptible cultivar, the expression of these same genes remained unchanged | MAPK signaling, Ca^2+^ signaling, ROS scavenging, and NBS-LRR | Yang et al., 2023 |
| DT – (Liaodou 14) and DS – (Liaodou 21) | Leaf | V2-V3 | 20% PEG 6000 for 24 hrs. | The leucine-rich repeat receptor-like kinases (LRR-RLKs) were uniquely downregulated in DS genotype but uniquely upregulated in DT genotype by drought stress | Photosynthesis, carbohydrate metabolism, lipid metabolism, cell wall organization, signaling pathways, and leucine-rich repeat receptor-like kinases | Li et al., 2024 |
| DS – (Liaodou 15) | Leaf | Early flowering stage | Withholding irrigation for 7 d for mild, 17 d for moderate, and 27 d for severe stress) and a control in field conditions | Drought stress induced the expression of *P5CS* and *PAO* genes that promote the accumulation of spermidine and proline, compared to the response in the control treatment | *PAO1*, *PAO4*, and *PAO5* | Wang et al., 2024a |
| DT – (Heinong 44) and DS – (Suinong 14) | Leaf | seed filling stage (R5, BBCH: 75) | Mild water deficit (60% pot soil water capacity) greenhouse experiment | The flavonoid metabolites of drought-tolerant variety increased, while those of drought-susceptible variety decreased | Flavonoid and isoflavone biosynthesis pathways | Xu et al., 2024 |

**Supplemental Table 3.** Summary of Proteomics Studies Revealing Soybean Responses to Drought and Osmotic Stress

| **Genotype / Variety** | **Tissue** | **Method / Technique** | **Number of DEPs Identified** | **Key Identified Proteins** | **Major Findings** | **Affected Metabolic Pathways / Cellular Functions** | **References** |
| --- | --- | --- | --- | --- | --- | --- | --- |
| Enrei | Root | 2-DE/ MALDI-TOF MS | 37:  19↑ & 18↓ | caffeoyl-CoA-O-methyltransferase and 20S proteasome alpha subunit A | PEG-induced osmotic stress alters root proteins that regulate early stress responses in soybean. | osmotic stress, cell structure, metabolism, secondary metabolism | (Toorchi et al., 2009) |
| Taegwang | Root | MALDI-TOF MS | 28  5↑ & 21↓ | glycerol kinase, arogenate, prephenate dehydratase, phloem serpin | Soybean roots activate novel proteins to maintain water balance and protect cells under drought stress. | carbohydrate and nitrogen metabolism, control of ROS, secondary metabolism, defense, signaling | (Alam et al., 2010) |
| Enrei | Leaf, roots, hypocotyls | 2-DE/Nano LC/MS | Leaf: 18-  9↑ & 9↓  Root: 11-  8↑ & 3↓  Hypocotyl:12-  7↑ & 5↓ | actin isoform B, hsp 70, methionine synthase Enolase, ascorbate peroxidase | Roots are the most drought-responsive organ, and methionine synthase is a key drought-response protein in soybean. | metabolism-related proteins, protein  synthesis | (Mohammadi et al., 2012) |
| Enrei | Plasma  membrane | LC MS/MS | 86:  11↑ & 75↓ | Calnexin,  H^+^-ATPase  phototropin, protease inhibitor | Osmotic stress activates plasma membrane H⁺-ATPase through coordinated regulation of activator and suppressor proteins, enhancing ion transport for stress tolerance. | osmotic stress responsive, protein folding and protein synthesis, transporter | (Nouri et al., 2012) |
| Tanggamus, Nanti, Seulawah and Tidar | Leaf | SDS-PAGE / Western blotting | 13 | Dehydrin | Only drought-tolerant plants showed adaptive protein changes and maintained dehydrins under drought stress. | Oxidative stress | (Arumingtyas & Savitri, 2013) |
| Surge and Davison | Leaf | 2D-DIGE/ MALDI-TOF MS/ TOF/TOF tandem MS | 44  16↑ & 28↓ | EF-Tu, glutamine synthetase, HCF136, stromal 70 kDa HSP protein | Higher EF-Tu and other stress-related proteins in soybean leaves enhance tolerance to drought and heat by protecting photosynthesis and cellular function. | RuBisCO regulation, electron transport, Calvin cycle, and carbon fixation | (Das et al., 2016) |
| Enrei | Root | Nano LC/MS | 643  67↑ >2fold | peroxidase and aldehyde dehydrogenase | Peroxidase and aldehyde dehydrogenase support soybean recovery after drought stress | hormone metabolism,  cell wall, secondary metabolism | (Khan & Komatsu, 2016) |
| Jawahar Soybean-335 | Leaf:  Vegetative & Flower stage | MALDI-TOF MS | Veg:41  33↑ & 8↓  Flo:41-  35 ↑& 6↓ | RuBisCO activase, chl a/b binding protein, ATP synthase, Amino transferase | Salicylic acid-maintained photosynthesis and carbon metabolism proteins, enhancing drought tolerance in soybean. | Carbon metabolism, Amino acid metabolism, Protein synthesis, assembly and degradation, Redox signaling | (Sharma et al., 2018) |
| Jawahar Soybean-335 | Pod walls | MALDI-TOF MS | 27:  19↑ & 4↓ &  4= | 14-3-3 protein, MAP kinase 3, Rubisco activase, Ferritin | Proteomics revealed that soybean pod walls respond rapidly to potassium iodide-simulated drought, activating early drought-tolerance mechanisms. | stress signaling / regulation, protein folding / stabilization, redox homeostasis | (Sengupta et al., 2019) |
| GN-2032, GN-3074 | Leaf | 2-DE/LC MS/MS | GN3074:14-  7↑ & 7↓  GN-2032: 12  6↑ & 6↓ | Rubisco Activas, chlorophyll a–b binding protein, trypsin inhibitor, RUBISCO, Ascorbate peroxidase | Drought-tolerant soybean preserves stress signaling and metabolism proteins, supporting stay-green under stress. | Photosynthesis, defense mechanisms, photorespiration, respiration, metabolism process | (Yahoueian et al., 2021) |
| Jiyu47 | Root | LC-MS/MS | 468:  144↑ & 324↓ | cellulose synthase; G-1-PG, sucrose synthase | Soybean roots rapidly adjust proteins involved in metabolism and glutathione-mediated antioxidant defense to cope with drought stress. | carbohydrate metabolism, osmotic regulation, antioxidant defense system | (Zhou et al., 2022) |

**Supplemental Table 4.** A summary of the literature on the application of metabolomics in soybean drought tolerance research.

| **Sample** | **Technique** | **Objective** | **Ref.** |
| --- | --- | --- | --- |
| [Drought-resistant] *Glycine soja* Siebold & Zucc. (Huinan06116)  [Drought-sensitive] *Glycine max* (L.) Merr. (Jinong24) | GC-MS (Untargeted metabolomics) | To identify key compounds and pathways that contribute to drought tolerance in wild and cultivated soybeans | (Fu et al., 2020) |
| *Glycine max* [Drought-resistant] NA5009RG [Drought-sensitive] DM50048 | ^1^H NMR (Untargeted metabolomics) | To evaluate the physiological and metabolic responses of drought-tolerant and sensitive soybean genotypes to drought stress and identify stress- or genotype-specific metabolite changes | (Silvente et al., 2012) |
| *Glycine max* [Drought-resistant] Overexpressed *ABF3* (Kwangan) [Drought-sensitive] Wild type (Kwangan) | GC-MS LC-MS (Untargeted metabolomics) | To investigate the metabolic changes and responses in transgenic soybeans overexpressing ABF3 under drought conditions | (Nam et al., 2019) |
| *Glycine max* [Drought-resistant] Desafio 8473 RSF, 7739 M IPRO  [Drought-sensitive] NS7209 IPRO, NS7011 IPRO | GC-MS LC-QTOF-MS (Untargeted metabolomics) | To examine the metabolic, physiological, and anatomical responses of four soybean cultivars to water deficit and high temperature stress, individually and combined, and to identify biomarkers highlighting the unique mechanisms of acclimation to combined stress | (Vital et al., 2022) |
| *Glycine soja* [Drought-resistant] Tongyu03611 [Drought-sensitive] Huinan06116 | GC-MS (Untargeted metabolomics) | To uncover the mechanisms responsible for drought tolerance in drought-tolerant wild soybean | (Wang et al., 2019) |
| *Glycine max* [Drought-resistant] Heinong 44 (HN44) [Drought-sensitive] Heinong 65 (HN65) | LC-MS (Untargeted metabolomics) | To investigate the amino acid metabolism in the roots of drought-resistant and drought-sensitive soybean varieties under drought stress | (Wang et al., 2024b) |
| *Glycine max* [Drought-resistant] Heinong 44 (HN44) [Drought-sensitive] Heinong 65 (HN65) | LC-MS (Untargeted and targeted metabolomics) | To identify differential metabolites and examine physiological indexes in soybean leaves under drought stress | (Wang et al., 2022c) |
| *Glycine max* [Drought-resistant] transformed 35S::BiP-4 [Drought-sensitive] wild type | GC-TOF-MS LC-MS (Targeted metabolomics)  2DE-nanoLC-MS (Proteomics) | To examine proteomic and metabolic profiles of BiP-overexpressing and untransformed soybeans under drought to understand their acclimation mechanisms | (Coutinho et al., 2019) |
| *Glycine soja* [Drought-resistant] Tongyu06311 [Drought-sensitive] Huinan06116 | GC-TOF-MS (Untargeted metabolomics)  RNA-Seq (Transcriptomics) | To uncover how the regulation of hydrogen cyanide and γ-aminobutyric acid contribute to drought resistance of wild soybeans | (Zhang et al., 2023) |
| *Glycine max* [Drought-resistant] Lin Xian Xiao Huang Dou [Drought-sensitive] Jin Da Zao Huang | LC-MS (Untargeted) RNA-Seq (Transcriptomics) | To characterize the molecular basis of drought tolerance in the soybean using multi-omic approaches and propose a regulatory mechanism and adaptive traits applicable to cultivated soybeans | (Zhao et al., 2021) |
| *Glycine max*  [Drought-resistant] PI 603535, G21-2318, G21-2322 [Drought-sensitive] Benning, G21-2245, G21-2334 | LC-MS (Pathway-based metabolomics) | To characterize differentially expressed metabolites between drought-tolerant and susceptible genotypes and identify metabolic reprogramming strategies involving primary and secondary metabolism. | (Lee et al., 2026) |

^*^ GC-MS: gas chromatography/mass spectrometry; NMR: nuclear magnetic resonance; LC-MS: liquid chromatography-mass spectrometry; LC-QTOF-MS: liquid chromatography-quadrupole time of flight-mass spectrometry; GC-TOF-MS: gas chromatography-time of flight-mass spectrometry; 2DE-nanoLC-MS: two-dimensional gel electrophoresis-nanoflow liquid chromatography-mass spectrometry; RNA-Seq: RNA sequencing

**Supplemental Table 5.** List of potential metabolite markers selected from soybean drought tolerance research (Silvente et al., 2012; Coutinho et al., 2019; Nam et al., 2019; Wang et al., 2019, 2022b, 2024b; Fu et al., 2020; Zhao et al., 2021; Vital et al., 2022; Zhang et al., 2023).

| *Primary metabolites* |  |
| --- | --- |
| Sugars and sugar alcohols | Allose, fructose, fucose, galactinol, glycerol, glucose, glucose-1-phosphate, glycerol, inositol, lactose, maltose, mannitol, mannose, meliobiose, *myo*-inositol, pinitol, raffinose, ribose, sorbitol, sucrose, xylitol, xylose |
| Amino acids | Alanine, arginine, asparagine, aspartic acid, cysteine, glutamic acid, glycine, histidine, isoleucine, leucine, lysine, methionine, phenylalanine, proline, serine, threonine, tryptophan, tyrosine, valine, γ-aminobutyric acid |
| Fatty acids | Arachidic acid, linoleic acid, linolenic acid, octadecanoic acid, oleic acid |
| Organic acids | α-ketoglutaric acid, citric acid, fumaric acid, glucoheptonic acid, gluconic acid, gluconic lactone, glutaric acid, glyceric acid, malic acid, malonic acid, nicotinic acid, pyroglutamic acid, saccharic acid, shikimic acid, succinic acid, tartaric acid, threonic acid, 4-guanidinobutyric acid |
| *Secondary metabolites* |  |
| Flavonoids | Apigenin, kaempferol |
| Phenylpropanoids | Caffeic acid, ferulic acid |
| Phytohormones | Abscisic acid, jasmonic acid, salicylic acid |
| Others | Trigonelline |
